# Bioethical considerations in deploying mobile mental health apps in LMIC settings: insights from the MITHRA pilot study in rural India

**DOI:** 10.3389/fdgth.2026.1634669

**Published:** 2026-04-10

**Authors:** Yesenia Navarro-Aguirre, Bharat Kalidindi, Ramakrishna Bhooma Goud, Johnson-Pradeep Ruben, Abijeet Waghmare, Dhinagaran Devadass, Tony Raj, Krishnamachari Srinivasan, Amritha Bhat

**Affiliations:** Department of Psychiatry and Behavioral Sciences, University of Washington, St. John’s Research Institute, Seattle, WA, United States

**Keywords:** cultural alignment, mobile mental health app, informed consent, participant autonomy, privacy and data protection

## Abstract

**Introduction:**

In India, untreated depression among women contributes significantly to morbidity and mortality, underscoring an urgent need for accessible and ethically grounded mental health interventions. Mobile health (mHealth) tools offer scalable solutions; however, their implementation in low- and middle-income country (LMIC) settings raises important bioethical considerations.

**Methods:**

This study was conducted at the conclusion of a pilot randomized controlled trial evaluating the MITHRA app (Multiuser Interactive Health Response Application), designed for depression screening and treatment among women participating in self-help groups (SHGs) in rural Karnataka, India. Two focus-group discussions were conducted with intervention participants to explore ethical dimensions of app use, including technological proficiency, privacy, informed consent, connectivity, accessibility, and gender-specific interactions. Transcripts were analyzed using thematic coding to identify recurring patterns.

**Results:**

Participants preferred a hybrid care model combining mobile app use with human interaction. Technological proficiency varied, and participants demonstrated uncertainty regarding mental health app functionality and limited understanding of privacy policies. In the collectivist cultural context of rural India, autonomy and informed consent were often expressed relationally, shaped by family and community dynamics rather than individual decision-making alone.

**Discussion:**

These findings highlight the need to tailor digital mental health interventions to user preferences and local sociocultural contexts. Ethical implementation in rural LMIC settings requires enhanced transparency around data use, culturally aligned consent processes, and integration of ethical, technological, and relational considerations to improve accessibility, trust, and acceptability.

## Introduction

Mobile health (mHealth) technologies offer scalable, accessible solutions to mental health care in low-resource settings. By addressing barriers like stigma, limited infrastructure, and the scarcity of mental health professionals, mHealth tools can bridge significant gaps in care. In India, mobile health (mHealth) interventions began gaining traction in the mid-2010s, especially through government and NGO efforts in maternal, child, and reproductive health (1, 2). For example, a 2018 systematic review covering studies published from January 1997 to June 2017 by Bassi et al., catalogued over 200 mHealth and telemedicine initiatives by that time, many focusing on preventive care and health education, but only a small number specifically addressed mental health in rural zones (1). More recent work shows incremental growth in mental health app availability and awareness among Indian users, though challenges remain around privacy, evidence base, interface design, and accessibility for low-literacy populations (2, 3).

Despite these advances, the implementation of mHealth innovations raise ethical concerns, particularly around informed consent, data privacy, and equitable access, especially in low- and middle-income countries (LMICs) with digital divides, i.e., gaps in access to technology and digital literacy (4–6). These concerns are particularly critical when implementing technologies among vulnerable populations, such as rural women, who face compounded challenges of limited agency, digital illiteracy, and systemic inequities.

In rural India, mobile health solutions often align better with collectivist models of technology use, such as shared or community-accessible devices, rather than the individual ownership model prevalent in high-income settings (4). The Multiuser Interactive Health Response Application (MITHRA) exemplifies both the promise and challenges of mHealth interventions. Designed to support women in rural India who suffer from low to moderate depression, the app tailor's mental health interventions based on participants' depression severity as measured by Patient Health Questionnaire (PHQ-9) scores. It was developed using participatory design methods and aims to address the cultural and technological needs of the target population (7, 8). Regionally, among women in southern Karnataka, a community-based survey reported common mental disorders in 5.7%, including depressive disorders in 4.6%, underscoring the need for accessible detection and care pathways that mHealth could help address (9). This study addresses that gap by examining MITHRA's implementation through a bioethical lens. We define bioethical considerations as including Informed consent; Privacy and data protection; Participant autonomy; Equitable access and Cultural alignment.

By exploring how women in rural India perceive these issues, we ask: What are the ethical considerations in using mobile mental health apps to detect and treat depression in underserved populations? To address this question, we present an empirical case illustration from South Asia using qualitative focus-group data collected from participants in the MITHRA pilot randomized controlled trial conducted in rural Karnataka, India. Rather than offering a purely conceptual ethical analysis, this paper grounds bioethical principles in real-world implementation experiences, examining how autonomy, privacy, and equity are understood and negotiated within a collectivist context characterized by shared device use, variable digital literacy, and relational decision-making. This focus on cultural alignment is essential to ensure that digital interventions are not only scalable, but also ethically sound, trustworthy, and responsive to local norms and lived realities.

This paper does not propose a new ethical framework. Instead, it applies established bioethical principles to empirical findings from a real-world mHealth deployment in rural India to identify practical ethical risks and implementation strategies. In doing so, we offer actionable considerations for developers, clinicians, and health systems seeking to implement digital mental health tools in low-resource settings.

## Materials and methods

This study was conducted under the ethical oversight of the Institutional Review Board at the University of Washington and the Institutional Ethics Committee at St. John's Medical College.

This supplemental project is part of a larger study titled “Multiuser App for the Detection and Treatment of Depression in Women's Self-Help Groups: A Pilot Randomized Controlled Trial.” This two-year regional mixed methods study aimed to evaluate the feasibility, acceptability, and preliminary effectiveness of the MITHRA app; a mobile health app specifically developed to address depression among women in rural India (9). The pilot RCT, conducted in Anekal taluk, Karnataka, randomized Self-Help Groups (SHGs) across 10 community-based organizations to either app-based treatment or enhanced usual care, enrolling 85 women aged 20–60. Of these, 49 participants were in the intervention arm, with over half actively engaging and completing the assigned modules on the MITHRA app.

### Ethical considerations

To ensure accessibility for participants with limited literacy, all components of the MITHRA app were designed with audio-visual content and icon-based navigation. Trained facilitators introduced participants to the app through live demonstrations and verbal instructions during SHG meetings, ensuring that even those who could not read were able to understand and engage with the intervention. The app was developed entirely in Kannada, the native language of participants in Anekal taluk, and all in-app content including audio, videos, and text was translated for dialect and cultural relevance. Instructions were also reinforced during focus group sessions and via community health workers to support comprehension and sustained use.

Participants who were part of the intervention arm of MITHRA RCT were chosen for this sub study. Informed consent was signed by all these participants and for those who were illiterate, the consent form was verbally explained aloud in Kannada, the local language used for verbal comprehension in the presence of an impartial witness, and thumbprint was obtained on the consent form ensuring that the participants understood the study, its risks, benefits, and their right to decline participation or withdraw at any time without penalty. Confidentiality was maintained by de-identifying transcripts and storing data in encrypted password-protected systems.

Given the collectivist cultural context of rural India, where health decisions are often relational, it was understood that participants may have consulted family members such as spouses or elders prior to enrolling in the study, even if this was not directly documented. This reflects a broader cultural model of relational autonomy, in which decision-making is shaped by family dynamics, mutual obligation, and shared responsibility rather than individual independence alone (10, 11). Community health workers and SHG leaders played a central role in facilitating trust and social acceptance of the intervention, aligning with relational ethics frameworks that emphasize social ties and moral accountability within the community (12–14).

### Focus group discussions

To further investigate the ethical dimensions of using mHealth technologies, the study team conducted two focus group discussions (FGDs) in August 2023, drawing participants from the intervention arm of the RCT. Two women's self-help groups, denoted as SHG1 and SHG2, served as the primary locations for participant recruitment in this sub-study*.* Sixteen women, with a mean age of 36.6 years (SD = 5.7, range 28–48), were recruited, with eight participants per focus group. Participants were clustered based on their PHQ-9 scores, which ranged from 0 to 13, with a mean score of 4.0 (SD = 3.7). The groups included women with no depression (PHQ-9 < 5), mild depression (PHQ-9 scores 5–10), and moderate depression (PHQ-9 scores 10–15) who had completed the MITHRA intervention. Participants from SHG1 averaged 7.9 years of education, while those from SHG2 averaged 4.9 years. The most common occupation among participants was unskilled manual labor, and the predominant family structure was nuclear. SHG1 participants had an average of 3.5 adult family members and 1.2 children per household, with an average monthly family income of ₹24,000. In comparison, SHG2 participants had slightly more adult family members (4.0) and 1.0 children per household, with a lower average monthly family income of ₹20,000. This diverse sampling allowed the exploration of multiple perspectives.

We conducted two 90-minute focus groups, using semi-structured interviews to explore participants' experiences and perceptions of the MITHRA app. The interview guide included open-ended questions that explored participants preferences for digital vs. in-person mental health support, accessibility challenges, understanding privacy and data sharing, and perceptions of consent. Additional topics included technological familiarity, scheduling conflicts, gender-based access issues, and stigma. Questions were developed based on the Unified Theory of Acceptance and Use of Technology (UTAUT), which guided domains on performance and effort expectancy, social influence, and facilitating conditions (15). Ethical probes were structured by the Four Principles of Biomedical Ethics, autonomy, beneficence, non-maleficence, and justice (16).

During the focus groups, the research team occasionally needed to clarify certain questions to ensure participants understood and could actively engage in discussions. This approach helped improve the quality of the dialogue but may have influenced participants' responses by shaping their interpretations, particularly on bioethical issues. Although participants did not explicitly describe concepts like privacy or autonomy, their responses often reflected collective patterns of decision-making, such as consulting family members or discussing shared phone use, indicating that ethical engagement occurred within relational and community-based frameworks and is in alignment with earlier literature on women's autonomy and agency (14).

The data were analyzed through thematic analysis by two independent coders who reviewed all transcripts together. One coder was a member of the data collection team, providing contextual insights, while the other was a research coordinator offering an external perspective. As both coders worked collaboratively on all transcripts, inter-rater reliability was not calculated. We conducted a thematic analysis that followed a primarily deductive approach grounded in prewritten themes derived directly from the questionnaire and interview guide. These predetermined themes and subthemes structured the analysis from the outset, ensuring alignment between the data collection instrument and analytic framework. As transcripts were reviewed, additional codes were generated inductively when participants introduced ideas that extended or nuanced the original categories. Both coders collaboratively refined the codebook to incorporate these emergent codes while maintaining consistency with the predetermined framework. This approach allowed the analysis to remain systematic and anchored to the study's research questions while still flexible enough to capture unanticipated insights from participants. Table 1 presents the final set of themes, subthemes, and corresponding codes developed through this process.

**Table 1:** Themes, subthemes, and definitions from focus groups.

Theme	Sub themes	Associated quotes
**Mobile Device Utility:** Views on the practical uses of mobile devices in daily life, such as communication, transactions, accessing health info, and task management.	**Personal Use Purpose—**Specific reasons individuals use smartphones outside of communication.	*P: “Not only that, but you can search that in google if you put one product, 10 products are displayed and we can select from that, oh it will good for me”*
**Connectivity and Accessibility:** Technological and environmental conditions shaping participants' ability to access and engage with the app.	**Daily Access:** The extent to which users can access their mobile phones.	*P: “Husband has the phone, after he comes back from the work if he gives will use it”*
		*P: “I used it 4–5 times”*
	**Mental Health App Usage Frequency:** How often users engage with Mental Health apps.	*P: “I used it 4–5 times”*
	**Scheduling Concerns:** Challenges related to planning times for app usage.	*RC: “What is the reason you don't have enough time to use the app?.”*
		*P: “Because will go to work know sir”*
	**Data Cost Concerns:** Issues related to mobile data costs when using apps.	*P: “Currency will be a problem, if you install it in your mobile phone currency will be a problem.”*
**Technological Competence:** The level of digital literacy and comfort influencing participants' engagement with the app.	**MH App Understanding:** Understanding of what a mobile mental health app is.	*P: “We feel that MITHRA APP is like our friend, few things which couldn't be shared outside we could share it here, some information which we were unaware, could get through MITHRA APP.”*
		*P: “From MITHRA APP we learnt how to get rid from depression. How to maintain hygiene (wash hands and eat) when we get angry we get suicidal thoughts, so MITHRA APP helped us to come out of it.”*
	**Perceived Benefits:** The advantages seen by users and their communities from MH apps.	*P: “From MITHRA APP we learnt how we should be good with your friends, how should you talk to others, how you should solve your family problems”*
	**Knowledge of App Updates:** Knowledge about app updates and how to carry them out.	*RC: “Have any clue of what the update means?”*
		“*Sir to understand that we should be educated that but we are not that educated”*
	**App Functionality:** Knowledge of the features and operations of the app, ensuring optimal utilization of its functionalities.	*P: “I did not know how to play it so I took help from others and watched it”*
	**App Usage:** Understanding how to use the app, understanding its primary objective.	*P: “MITHRA APP Talks about mental problems”*
**Stigma and Privacy:** Social stigma and confidentiality that shape participants' willingness to engage with digital mental health tools.	**Device Sharing a & Privacy:** Patterns of sharing devices and concerns about privacy.	*RC: “We installed our APP in your phone, will you use it, it doesn't matter if your husband see it, do you watch with them?”*
		*P: “Yes we will”*
	**Perceptions on Data Access:** Feelings about others accessing personal app data.	*RC: “You know where the information will be passed? and what will happen?”*
		*P: “I don't know sir, you have to tell me sir”*
	**Community Interactions through the app:** Willingness and preferences in interacting with fellow users or participants.	*P: “So we don't know many things and we get to know about from them and whatever they don't know they get to know from us”*
	**Comfort level:** Comparing the comfort level of discussing personal issues with a professional vs. an app.	*P: “It is better to have a face-to-face interview with the doctor”*
**Gender Interactions:** The influence of gender roles, family dynamics, and social expectations on participants' access to and use of the digital intervention.	**Primary Device User:** Identifying who mainly uses the mobile device in households.	*RC: “Who mainly uses the phone”*
		*P: “My husband sir”*
	**Partner Awareness and Attitude:** Feelings and concerns related to partners knowing about app usage.	*RC: “So Now in APP you have to participate in several activities, is that fine your husband watches those activities.”*
		*P: “No problem”*
	**Gendered Sharing:** Comfort levels in sharing app-related activities with different family members.	*RC: “Can you share your MITHRA experience or mobile phone usage experience with your family members?”*
		*P: “Yes will share”*
	**Partner Support:** The degree of support from partners in the context of app usage and participation.	*RC: “You are afraid that the husband will object?”*
		*P: “No, no”*
		*RC: “You are worried that your husband will take your phone and use it”*
		*P: “I'll show this to my husband. Not scared”*

To ensure comprehensive and transparent reporting, this study adheres to the Standards for Reporting Qualitative Research (SRQR) (17) and aligns with the COREQ (Consolidated Criteria for Reporting Qualitative Research) checklist. Key elements including researcher roles, setting, participant selection, and data analysis.

Ethical approval for this study was obtained from the Institutional Review Board at the University of Washington (STUDY00010415) and the Institutional Ethics Committee at St. John's Medical College (IEC Study Ref. No. 184/2020). The study was conducted in accordance with ethical guidelines for research involving human participants. There were no adverse events, and no participants reported suicidal ideation or required a referral to the Primary Health Center per protocol.

## Results key themes and findings

### Mobile device utility

Participants often discussed the broad utility of mobile devices in their everyday lives, focusing on their phones' general benefits and diverse functions. **Mobile Device Utility** emerged as a central theme centered on using mobile phones for practical, everyday tasks. For instance, one participant explained, “During emergency if somebody needs money, we can send money through PhonePe (digital payment platform primarily used in India) or Google Pay.” This highlights the vital role mobile phones play in financial transactions, particularly in rural areas where access to physical banking services may be limited. Beyond financial transactions, participants also used their phones for various other tasks, such as shopping, entertainment, and accessing general information, showcasing the adaptability and necessity of mobile devices in these communities.

Participants also shared insights into their **Personal Use** of smartphones outside of communication. This theme reflects the various ways participants use their mobile phones beyond basic phone calls or messaging. Many participants cited health-related reasons as key motivations for using their phones. One participant mentioned, “It can be used to get information from doctors about health-related issues through mobile.” This suggests that mobile phones serve as a tool for health management, giving users access to medical advice, health information, and updates from healthcare providers. These responses highlight the multifunctionality of smartphones in the participants' lives and their potential to serve as a gateway to mental health services.

#### Connectivity and accessibility

The Mental Health App Usage Frequency sub-theme explored how often participants engaged with the MITHRA app. Usage patterns varied, with some participants using the app regularly, while others interacted with it less frequently. One participant shared, “I used it 4–5 times. Used for 4–5 days.” This response reflects a level of engagement that varied across participants. Some participants were able to integrate the app into their weekly routines, while others struggled to use it consistently. Daily access to a mobile phone shaped these patterns, as some users had predictable opportunities to use a device while others had more limited or shared access. Scheduling concerns—such as finding uninterrupted time or aligning use with other responsibilities—also influenced consistency, with engagement tending to cluster in windows that fit daily routines.

#### Technological competence

Participants generally grasped what the MITHRA app was for recognizing problems, coping strategies, and everyday health routines reflecting the MH App Understanding sub-theme; this sense of purpose appeared to boost confidence with basic operations, though it indicated comfort and motivation more than advanced skills. In practice, consistent with App Usage, most users could log in and watch videos after initial setup, but many relied on daughters, peers, or facilitators to start playback or troubleshoot, and only a small subset reported fully independent use; transferring skills to personal phones (e.g., downloading apps) was uncommon, indicating that most participants used only core app functions. Understanding of app “updates” was notably weak under Knowledge of App Updates. While a few associated updates with faster performance, most were unsure what an update prompt meant until it was explained.

#### Stigma and privacy

Most participants did not fear others discovering their use of the app and, in several households, welcomed spouses or family to watch alongside; however, everyday privacy was constrained by device control and access logistics (e.g., phones held by husbands, turn-taking), indicating that who controls the phone often determines who controls privacy. The sub-theme of Comfort Level focused on participants' varying degrees of ease in discussing personal issues, especially mental health concerns, through a mobile app as opposed to traditional in-person interactions with a healthcare professional. Many participants expressed a preference for face-to-face interactions, perceiving them as more personal, trustworthy, and effective for sensitive topics. As one participant put it, “It is better to have a face-to-face interview with the doctor.” This preference for direct interaction suggests that while participants may be open to using mobile devices for certain health-related tasks, they still find greater comfort in having personal, face-to-face conversations when it comes to discussing mental health. Additionally, the involvement of a research coordinator was mentioned by some of the participants, the research coordinator provided critical support by explaining app features, addressing participants' questions, and facilitating their understanding of the app's content.

#### Gendered interactions

Across these sub-themes, participants described household patterns that shaped when and how they accessed mobile phones. Under Primary Device User, smartphones were often managed by husbands (and sometimes children); so many participants used a shared device when it was available commonly in the evening while a subset reported personal phones. In Gendered Sharing, turn-taking and routine household responsibilities influenced access windows and session length, with some noting limited uninterrupted time even on non-workdays. Partner Support was frequently described as neutral to positive: most reported no objection from husbands and, in several cases, encouragement to use or share the app content. In Partner Awareness & Attitude, partners were often aware and at times co-viewed materials; participants generally did not anticipate discomfort if spouses saw the app, and some framed shared viewing as helpful for mutual understanding.

## Discussion

### Privacy, confidentiality, and informed consent in shared-device contexts

Building on the empirical findings, we interpret participants' experiences through a bioethical lens, with particular attention to privacy, autonomy, and informed consent. The ethical framework underpinning the MITHRA app was designed to address privacy and informed consent, utilizing biometric logins and personalized access measures to secure user data. This aligns with global recommendations for ethical mHealth design emphasizing user autonomy, data protection, and transparency (5, 6). However, in collectivist contexts like rural India, privacy and autonomy are often practiced and understood differently. Decision-making tends to be relational, shaped by family dynamics, community norms, and the realities of shared device use (10, 11). Rather than being rooted in individual independence, women's health decisions are frequently negotiated within social networks, reflecting a collectivist ethic that has been well documented in LMIC settings (11, 14).

Despite these efforts, some participants expressed confusion about the app's privacy policies, which suggests that additional education and transparent communication may be necessary to ensure users fully understand and trust the app's data security measures. While privacy concerns were not a predominant theme, this may reflect different cultural interpretations of privacy rather than the absence of concern. In a setting where digital tools are shared and decisions are often made collectively; personalized security features may not always be seen as relevant or meaningful. This could contribute to the uncertainty some participants expressed. This observation underscores the importance of proactively addressing potential areas of uncertainty to enhance user confidence and engagement (6).

Many users did not fully understand how their data was being used or protected, pointing to a need for clear, accessible explanations as part of the onboarding process. Educational efforts must go beyond technical instructions to ensure users are aware of their rights and understand the importance of privacy, enhancing their confidence in the app. These findings are consistent with prior LMIC mHealth work indicating that comprehension, not merely the presence of consent materials drives trust and sustained engagement (1, 2).

### Technological competence, access, and the ethics of digital inclusion

The focus groups revealed that the MITHRA app needs to integrate face-to-face sessions with MH professionals and more comprehensive onboarding to effectively meet the needs of its users. The findings highlight how variable technological competence and shared device use shape engagement with mHealth tools. This foundation of mobile phone usage implies that participants were familiar with technology in general, although using phones for mental health purposes was less common and presented a distinct set of challenges. The practice of device sharing within households further complicates technological competence, as shared access may limit opportunities for individual users to develop familiarity and confidence with the app (4). This inconsistency highlights the necessity for enhanced onboarding processes that not only introduce users to the app but also offer ongoing guidance and support to build confidence in using this technology. Given that mobile phone use in many households is shared and shaped by social hierarchies, onboarding and support efforts must account for family roles and dynamics that influence access and control over devices. Additionally, incorporating human interaction, such as periodic check-ins with mental health professionals or community health workers, could bridge the gap for users who feel more comfortable with in-person support.

### Preferences for human support in blended care models

Participants expressed a range of comfort levels when discussing personal mental health issues, with many preferring face-to-face interactions over app-based communication. This preference suggests that digital mental health solutions should not function as standalone interventions but as part of a blended care model that includes direct human support. The involvement of a particular research coordinator was frequently cited as valuable in providing explanations and became an apparent link between digital and in-person care for some participants. Participants appreciated the research coordinator's guidance and often relied on her to help interpret app content, reinforcing the idea that a hybrid approach combining digital tools with human assistance is crucial for optimizing mental health care delivery in low-resource settings (7, 18). This finding underscores the importance of designing mHealth interventions that include or complement in-person care, especially in settings where cultural norms favor interpersonal communication.

Consistent with prior digital MH literature, brief scheduled touchpoints improve comprehension and adherence, for particularly sensitive content that users prefer to discuss in person (19). Operationalizing this blended model through early check-ins and an in-app “talk to someone” option can translate preference into practice (19).

### Operationalizing ethical safeguards in low-resource settings

**Figure 1:**
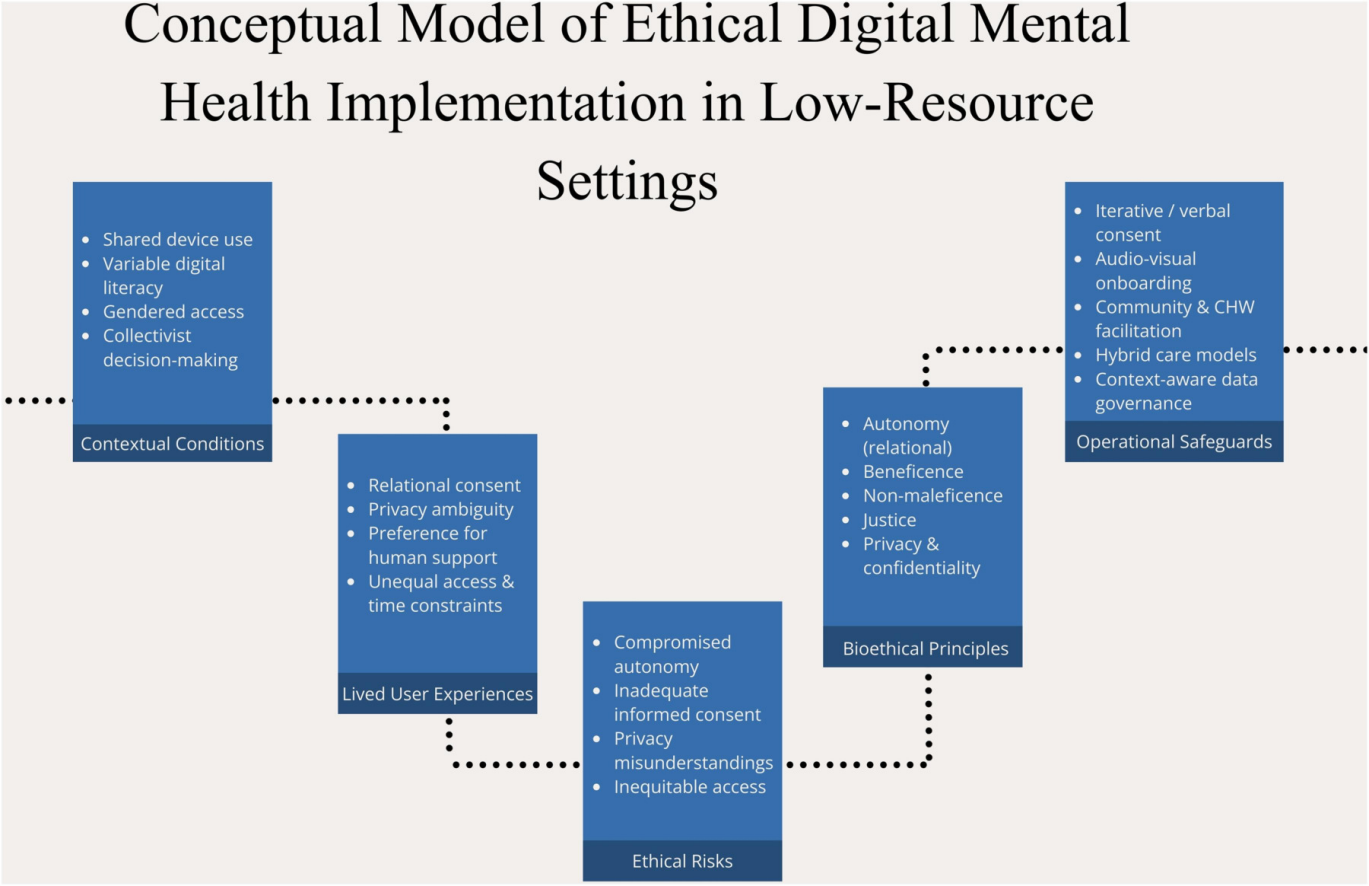
Conceptual model of ethical digital mental health implementation in low-resource settings.

Figure 1 synthesizes how contextual conditions shape lived user experiences, generate ethical risks, and inform operational safeguards in digital mental health implementation. Drawing on empirical findings from the MITHRA pilot in rural India, the figure illustrates how the four core bioethical principles (autonomy, beneficence, non-maleficence, and justice) function as an interpretive lens linking real-world use conditions to practical implementation strategies, with privacy and confidentiality treated as cross-cutting ethical domains shaping their application. Not all categories apply equally or directly to every finding; rather, the model visualizes how common bioethical constructs are used to interpret context-specific implementation challenges and translate them into actionable safeguards. The model does not propose a new ethical framework.

Moreover, the focus group findings suggest that without adequate onboarding and human support, there is a risk that technological barriers could exacerbate existing disparities in access to mental health care. A robust onboarding process, combined with ongoing human interaction, can help address these barriers, ensuring that digital health solutions are accessible, user-friendly, and tailored to meet the diverse needs of all users. By embedding these components, mental health apps like MITHRA can better support their users, improve engagement, and ultimately contribute to more effective and equitable mental health care in rural and under-resourced communities. Additionally, limited technological competence, particularly in communities where device sharing is common, further emphasizes the need for tailored solutions that address both technological and socio-cultural dynamics (5, 20, 21).

To operationalize these ethical considerations in practice, implementation strategies should translate bioethical principles into concrete safeguards that are feasible in low-literacy and shared-device environments. Consent should be treated as an ongoing process rather than a one-time event. This can include using verbal or audio-visual consent supports, incorporating brief “teach-back” checks to confirm understanding, and periodically reinforcing privacy and data-use concepts during onboarding and early engagement. This approach aligns with recent LMIC digital mental health implementation work, which emphasizes usability, cultural adaptation, and comprehension as key drivers of engagement (22).

Second, community engagement and facilitator-supported delivery can strengthen ethical implementation by leveraging trusted intermediaries (e.g., community health workers, women's group leaders, or clinic-based facilitators) to support onboarding, troubleshoot app use, and build trust through existing social and care relationships used in LMIC settings, including task-shared delivery through frontline workers (23).

Taken together, these findings illustrate how ethical concerns related to privacy, autonomy, and equity are not discrete but intersect through shared device use, gendered access, and variable digital literacy. Addressing these challenges requires implementation strategies that integrate ethical safeguards across technological, social, and clinical domains.

#### Strengths and limitations

One notable aspect of this study, which can be seen as both a strength and a limitation, was our deliberate decision not to explicitly define bioethics to participants in the context of the MITHRA app. Instead, we asked questions related to bioethical issues to gauge participants' natural understanding and perceptions. This approach yielded rich, authentic responses that offered valuable insights into participants' views on bioethics. However, it also limited our ability to assess participants' familiarity with these concepts comprehensively.

This study should be interpreted as a context-specific case illustration rather than representative of all “low-resource settings.” LMIC environments vary substantially in cultural expectations around privacy and autonomy, health-system infrastructure and referral pathways, legal and regulatory protections for digital data, and patterns of device access and digital literacy. Accordingly, the ethical challenges and mitigation strategies described here are most directly applicable to rural South Indian contexts similar to the MITHRA implementation and should be adapted in partnership with local stakeholders when applied elsewhere. During the focus groups, it became evident that some participants were confused by certain questions. The research team intervened to clarify the questions, which helped participants engage more effectively. However, this assistance also posed a limitation, as it may have influenced the participants' understanding and potentially affected the authenticity of their responses regarding their awareness of bioethical issues. Although the app was developed to reflect collective and relational understandings of decision-making, our analytic framework prioritized standardization and generalizability for broader LMIC mental health promotion, which required applying a Western bioethical paradigm. As such, this methodological tension between design and interpretation is acknowledged as a conceptual limitation of the study.

Additionally, another limitation of the study was that one of the coders was also a member of the data collection team for both the bioethics study and the larger MITHRA RCT. This dual involvement may have introduced bias, as their familiarity with the participants and prior data collection activities could have influenced their interpretation of the focus group transcripts.

## Conclusion

This study provides crucial insights that are essential for the ethical and effective implementation of the MITHRA app and other digital mental health interventions. Key considerations found through this research include technological competence, the importance of in-person support, user-friendly application interfaces, and robust informed consent processes. These elements are not just necessary for compliance but are fundamental to ensuring that the app meets the needs of its users in culturally sensitive and appropriate ways.

This study sheds light on the complexities of bioethical considerations in low-income settings, challenging preconceived notions about what is suitable for end users of mental health apps. The findings suggest that more attention needs to be paid to training users and developing tools that protect user privacy, while also assessing and enhancing technological competence, and allowing for hybrid mhealth and in person interventions. Such efforts are critical for fostering engagement, which in turn is vital for the success of interventions like MITHRA.

Future empirical research would benefit from going beyond traditional user participatory research by integrating bioethical considerations earlier in the app development process. When addressing bioethics, the focus should shift from merely understanding participants' ability to navigate and use technology to ensuring that they fully comprehend the nuances of the technology itself. This includes understanding where their data is stored, how their privacy is maintained, and whether they feel more comfortable with in-person care vs. digital interventions. In resource-limited settings, particularly where illiteracy is prevalent, these considerations become even more crucial. Researchers must not only engage participants but also serve as direct sources of support, guiding them through the complexities of the technology and ensuring that they grasp the implications of its use. This deeper level of engagement, combined with a proactive approach to bioethical considerations, ensures that participants are fully informed and comfortable with both the technology and the care it provides. By adopting a deliberative, ethically driven process from the outset, mHealth interventions can be more inclusive, equitable, and impactful, ultimately addressing the unique challenges posed by low-resource settings.

## Data Availability

The raw data supporting the conclusions of this article will be made available by the authors, without undue reservation.
